# HBV suppression by nucleos(t)ide analogues reduces PD-1 expression on liver-resident T cells

**DOI:** 10.1111/liv.70338

**Published:** 2025-11-01

**Authors:** Mireia García-López, Sabela Lens, Laura J. Pallett, Anna Pocurull, Thais Leonel, Ernest Belmonte, Ester García-Pras, Sergio Rodríguez-Tajes, Zoe Mariño, Maria Sàez-Palma, Concepción Bartres, Ariadna Rando-Segura, Francisco Rodríguez-Frías, Jonah Lin, Adam J Gehring, Mala K. Maini, Xavier Forns, Sofía Pérez-del-Pulgar

**Affiliations:** 1 Liver Unit, Hospital Clínic, University of Barcelona, IDIBAPS, CIBEREHD, Barcelona, Spain; 2 Institute of Immunity and Transplantation, Division of Infection and Immunity, University College London, London, UK; 3 Radiology Department, Centro de Diagnóstico por la Imagen (CDI), Hospital Clínic, University of Barcelona, Barcelona, Spain; 4 Liver Pathology Unit, Department of Biochemistry and Microbiology, Hospital Universitari Vall d’Hebron, Universitat Autònoma de Barcelona, CIBERehd, Barcelona, Spain; 5 Toronto Centre for Liver Disease, Toronto General Hospital Research Institute, University Health Network, Toronto, Ontario, Canada; 6 Department of Immunology, University of Toronto, Toronto, Ontario, Canada

**Keywords:** Hepatitis B virus, antiviral therapy, viral biomarkers, covalently closed circular DNA, immune response, PD-1

## Abstract

**Background & Aim:**

PD-1 expressing-T cells within the HBV-infected liver constitute a target of novel immunotherapeutics. Our aim was to investigate the impact of viral suppression on PD-1 expression on intrahepatic versus circulating lymphocyte populations from chronic hepatitis B (CHB) patients.

**Methods:**

22 CHB patients, 9 of them on nucleos(t)ide analogs (NUCs), had paired blood, liver fine needle aspirations (FNAs) and biopsies. A subset had a follow-up FNA after treatment initiation (n=4) or discontinuation (n=4). Intrahepatic (iHBV-DNA and cccDNA) and serum (HBV-DNA, HBsAg, HBcrAg and cirB-RNA) viral markers were quantified. Flow cytometry was used for immunophenotyping PBMCs and intrahepatic lymphocytes. An independent liver FNA scRNAseq dataset was used to consolidate our results.

**Results:**

PD-1 expression on tissue-resident memory CD8 T cells (T_RM_) correlated with both iHBV-DNA and cccDNA, as well as surrogate markers of cccDNA transcriptional activity (cirB-RNA and HBcrAg) in CHB patients with mild hepatitis. These associations were not reflected in circulating T cells. PD-1 expression intensity on CD8 T_RM_ was lower in NUC-treated than in naive patients, changes that were again not detectable in the circulation. Longitudinal analysis showed that viral load rebound induced by NUC discontinuation had the potential to drive re-expression of high levels of PD-1 on CD8 T_RM_. Conversely, therapy initiation and subsequent viral suppression reversed these changes. scRNAseq results further extended the profiling of these PD-1+CD8 T_RM_, showing a phenotype consistent with of bystander activation in response to subclinical liver damage.

**Conclusions:**

Intrahepatic viral markers correlate with PD-1 expression on global liver-resident T cells of CHB patients with mild hepatitis, with a reduction after prolonged NUC therapy and re-expression following treatment withdrawal.

## Introduction

Despite the existence of a prophylactic hepatitis B vaccine for more than 30 years, hepatitis B virus (HBV) infection is still a global health problem. Most recent WHO estimates indicate that more than 290 million people were living with chronic hepatitis B (CHB) in 2019, with 1.5 million new infections each year.[1] The natural history of chronic HBV infection is heterogeneous and depends on a dynamic interaction between the virus and the immune system of the host. Patients in the chronic hepatitis phase (both HBeAg-positive and HBeAg-negative) are usually treated with nucleos(t)ide analogs (NUCs) to prevent or at least slow the progression to fibrosis, cirrhosis, and hepatocellular carcinoma. However, despite effectively suppressing viral replication, NUCs have little effect on cccDNA, and HBsAg loss (functional cure) is only achieved in less than 5% of HBeAg-negative CHB patients.[2]The presence of a compromised immune response to the virus adds an extra hurdle to the pursuit of a cure for CHB.[3] Persistent exposure to viral antigens, together with the tolerogenic liver environment, contributes to the depletion and dysfunction of HBV-specific T cells. Hence, there is a need to advance immunotherapeutic strategies to restore T cell functionality and provide sustained immune control over the virus.

The analysis of T cells compartmentalised in the liver, the site of disease, is relevant to get a full picture of the immune response to HBV infection. Besides liver biopsy, fine needle aspirates (FNAs) have proved to be a reliable tool to sample immune populations within the liver. [4,5] Pallett et al. showed that a large proportion of HBV-specific CD8 T cell responses have a tissue-resident phenotype (CD69+CD103+CXCR6+), resulting in their compartmentalization in the liver.[6] These tissue-resident memory (T_RM_) CD8 T cells express high levels of the co-inhibitory receptor PD-1 and, in the context of HBV-infection, remain functional in terms of antiviral responses. Besides the bona fide CD8 T_RM_, a CD69+CD103- T_RM_-like population exhibiting an intermediate phenotype between the non-resident and the tissue-infiltrating has been described. Although their exact role is not clear, they may represent a pool of T cells lodged within the local microenvironment with the potential to mediate liver pathogenesis. Thus, it is considered that CD8 T cells in the HBV-infected liver play a dual role. On the one hand, increased frequencies of HBV-specific CD8 T cells are associated with better control of HBV infection.[7,8] On the other hand, their accumulation may exacerbate organ damage via the activation of bystander non-HBV-specific cells.[9]

Despite the fact that both HBV-specific and bystander CD8 T_RM_ express high levels of PD-1, a recent study suggests that cell-autonomous IL-2 production may limit their exhaustion thereby promoting antiviral effector functions.[10] In addition, IL-10 production by HBV-specific CD8 T cells upon antigen recognition enhances IL-2 responsiveness, thus promoting survival of effector CD8T cells.[11] Therefore, CD8 T_RM_ may enhance their survival and function and, indirectly, exert a relevant role in immune-regulation via the IL-2 and PD-1 axis.

Based on previous data, targeting the PD-1/PD-L1 axis seems a promising therapeutic strategy to restore virus-specific T cell responses and the liver immune tolerance.[12] Data from CHB patients showed that in vitro blockade of the PD-1 interaction with its ligand PD-L1 enhanced the antiviral function of both peripheral and intrahepatic HBV-specific T cells.[13,14] Results from a recent phase 1b clinical study showed that anti-PD-1 blockade resulted in a reduction of HBsAg levels (> 0.5 log IU/ml) in a limited proportion of virally suppressed HBeAg-negative patients.[15] Improved HBsAg reductions have been observed when combining a next-generation therapeutic vaccine encoding the inactivated polymerase, core, and the entire S region, and low dose nivolumab.[16] However, the full beneficial effect of therapeutic vaccines combined with PD-1/PD-L1 inhibition in CHB patients as well as predictors of response remain to be elucidated.

The fact that relevant responses may be enriched or compartmentalized in the liver, underscore the importance of sampling the liver to understand CHB pathogenesis and predict the effect of new therapeutic options. For this reason, we aimed at exploring the relationship between intrahepatic biomarkers of viral replication and the expression of the key immunotherapeutic target PD-1 in liver-resident T cells and its modulation by prolonged NUC-induced viral suppression. We observed a positive correlation between intrahepatic HBV-DNA and PD-1 expression in CD8 T_RM_ cells. Furthermore, the levels of PD-1 expression on CD8 T_RM_ were lower in FNAs from long-term virally suppressed patients than in treatment-naive patients, whereas NUC withdrawal appeared to have the potential to drive PD-1 re-expression on CD8 T_RM_. Finally, single cell RNA sequencing (scRNAseq) data from a separate cohort of patients starting NUC therapy was used to corroborate our findings, confirming decreases in PD-1 in transcriptionally defined tissue-resident CD8 T cell populations.

## Methods

### Patients and samples

This prospective study included 22 patients with CHB, 9 of whom were under long-term antiviral therapy with NUCs and 13 were treatment-naive. Exclusion criteria were advanced liver disease (F4 according to METAVIR by liver biopsy or previous diagnosis of cirrhosis), immunosuppressive therapy, hepatocellular carcinoma, or coinfection with HIV, HCV, or HDV.

At study enrolment, patients underwent an ultrasound-guided percutaneous liver biopsy and a fine-needle aspiration (FNA, 2 passes). Liver biopsies were divided into two parts, one for conventional histological analysis and the other part was stored in Allprotect Tissue Reagent (Qiagen, Hilden, Germany) at -80°C for molecular analyses. Follow up FNAs were also obtained between 6 and 18 months after stopping NUC therapy (n= 4 patients), or after starting antiviral treatment (n= 4 patients). Peripheral blood mononuclear cells (PBMCs) and serum samples were also collected at each visit.

### Viral serum parameters

HBeAg and anti-HBe were assessed by immunoassay using the Advia Centaur^®^ System (Siemens, Erlangen, Germany). Serum HBV-DNA was determined by real-time PCR using the cobas^®^ 6800 system (Roche Diagnostics, Manheim, Germany; LLQ <10 IU/ml). HBsAg was quantified using the ARCHITECT^®^ HBsAg assay (Abbott Laboratories, Chicago, IL, USA; LLQ <0.13 IU/ml). HBV core-related antigen (HBcrAg) levels were measured by chemiluminescent enzyme immunoassay using LUMIPULSE^®^ G1200 Analyzer (Fujirebio Europe, Gent, Belgium; LLQ <3 log U/ml) according to the manufacturer’s instructions. Circulating HBV-RNA (cirB-RNA) was assessed by real-time PCR (LLQ 10 cp/ml; linearity range 10 to 109 copies/ml on armored RNA template) using the Roche HBV-RNA investigational assay for use on the Cobas^®^ 6800/8800 Systems (Roche Diagnostics, Pleasanton, CA, USA). HBV genotype was determined by direct sequencing and phylogenetic analysis.[17]

### Virological tissue markers

Total nucleic acids were purified using the MasterPure™ DNA and RNA purification kit (Epicentre, Illumina, San Diego, CA, USA). Nucleic acid preparations were treated with RNase A and total DNA was quantified using the Qubit^®^ 3.0 Fluorometer (Invitrogen, Thermo Fisher Scientific, Waltham, MA, USA). Total intrahepatic HBV-DNA (iHBV-DNA) and cccDNA were determined as previously described.[17]

### Analysis of immune cell populations by flow cytometry

PBMCs were isolated by density gradient centrifugation using Ficoll Histopaque (Sigma-Aldrich, St. Louis, MO, USA) and intrahepatic leucocytes from FNAs as previously described.[5] Cell samples were suspended in RPMI 1640 medium (Thermo Fisher Scientific) supplemented with 10% heat-inactivated human serum (Sigma-Aldrich), 100 U/ml penicillin, 100 ug/ml streptomycin, non-essential and essential amino acids (Thermo Fisher Scientific) and 20 IU/ml rhIL-2 (Miltenyi Biotec, Bergisch Gladbach, Germany).

After overnight resting, cells were washed and stained with a fixable Live/Dead dye (Thermo Fisher Scientific) before incubation with saturating concentrations of surface mAbs diluted in 50% Brilliant stain buffer (BD Biosciences, San Jose, CA, USA) and 50% 1x PBS for 30 min at 4°C. Cells were fixed with Cytofix/Cytoperm (BD Biosciences) according to the manufacturer’s instructions. All samples were acquired on a BD LSR Fortessa flow cytometer and analysed using FlowJo v10 (BD Biosciences). A list of mAbs used for flow cytometry is provided in Supplementary Table S1. Gating strategy for global lymphocyte populations is shown in Supplementary Figure S2.

### Single cell RNA sequencing dataset

Paired single-cell RNA sequencing (scRNA-seq) data of FNAs derived from 5 CHB patients at baseline and after 24 weeks of TAF therapy previously published in Nkongolo S. et al. [6] were obtained from GEO (GSE216314).

Seurat version 4.0 was used for the following procedures. Prior to the analysis, quality control steps were performed. Cells were filtered by the number of genes detected (>300), the percentage of mitochondrial genes detected (<10% among all genes), the complexity of the cell (number of features divided by the number of counts, >0.8), the percentage of HBB genes per cell (<0.25) and the percentage of platelet genes per cell (<0.2). Only cells satisfying all five criteria were retained to construct the reference data. Cell cycle scores were used to determine if the samples were biased thereby. Additionally, uninteresting sources of variation within the data were removed (genes encoding ribosomal structural proteins, non-coding rRNAs, HBB, and genes expressed in <10 cells). CD8 T cells were filtered considering the markers used in the flow cytometry (CD3+, CD56 (NCAM1)-, CD19-, CD8+). After filtering, a total of 7,146 CD8 T cells remained for downstream analysis.

Data were normalized using the LogNormalize method (total UMI count for each cell was set to 10,000). The top 2,000 variable features were determined based on the variance stabilizing transformation function (FindVariableFeatures) with default parameters. PCA dimensionality reduction was performed on the normalized gene expression counts using RunPCA function. The first 15 principal components (based on the manual inspection of the elbow plot) were used for cell clustering (using the FindClusters function with resolution 0.2) and UMAP visualization (using RunUMAP). Then, clusters were annotated manually based on several criteria: (i) average expression of key marker genes in individual clusters, (ii) gradients of gene expression over the UMAP representation of the reference map, and (iii) literature of canonical markers.

The FindMarkers function was used to identify differentially expressed genes and the heatmaply R package to display some of the top genes as a heatmap. To identify the PD-1+ CD8 TRM population, a new subset within the CD8 T cells was performed by using the subset function. The DESeq2 R package was used to perform pseudobulk differential expression analysis between the baseline and follow-up samples. The paired t test was used to compare gene expression levels of specific genes within each patient between the 2 time points.

### Statistical analysis

Categorical variables are expressed as n (%) and quantitative variables as median and interquartile range (IQR). The Chi Square test was used to analyze relationships between categorical variables. For quantitative variables, paired comparisons were performed using the paired t test, and unpaired comparisons using the Mann-Whitney U test or Krustal Wallis for comparisons across more than two groups, with Dunn’s multiple comparisons test. For the correlative analysis between two quantitative variables, Spearman’s rank correlation coefficient test was used. Statistical significance was set at p<0.05. Statistical analyses were performed using either SPSS v20.0 (IBM, Chicago, IL, USA), GraphPad Prism v10 (GraphPad Software, Inc., CA, USA) or Origin 2021b (OriginLab Corporation, Northampton, MA, USA).

## Results

### Clinical and virological characteristics of the study cohort

Baseline clinical and virological characteristics of the patients included in the study are shown in Table 1 (grouped patients) and Supplementary Table S2 (individual data). Most patients were male (73%) with a median age of 46 years and had mild fibrosis (86% <F2).

Patients undergoing NUC therapy were HBeAg-negative and had undetectable viral load. The median treatment duration was 7 years (IQR 5-10) and most of them (78%) were treated with tenofovir. Treatment-naive patients had a median viral load of 5.2 Log IU/ml (IQR 3.4-8.4) and 5 of 13 (38%) were HBeAg-positive. Notably, 11 of the 13 treatment-naive patients (85%) had normal to mildly elevated ALT levels. HBsAg titres were similar between both groups of patients. Although the proportion of patients with detectable HBcrAg was similar between treated and untreated patients (56% vs 62%, respectively), treatment-naive patients showed higher HBcrAg levels compared to patients undergoing NUC therapy. CirB-RNA was detected in only 1 (11%) treated patient compared to 69% of treatment-naive patients. Regarding intrahepatic viral biomarkers, both iHBV-DNA and cccDNA were higher in the treatment-naive group and both parameters were significantly correlated with serum HBV-DNA, cirB-RNA and HBcrAg (Supplementary Figure S1). In agreement with previous reports,[17–19] no correlation was observed between HBsAg and HBcrAg and intrahepatic markers in patients under NUC therapy.

### PD-1 expression on CD8 T_RM_ is associated with HBV replication

First, we analysed the immune profiles of FNAs and compared them to peripheral blood from the same individual. Human liver tissue-resident CD8 T cells (T_RM_) were identified by the expression of two tissue retention molecules: CD69 (S1PR1 antagonist, preventing tissue egress) and CD103 (integrin αE that binds E-cadherin) (Supplementary Figure S2). Non-resident tissue-infiltrating or “recirculating” CD8 T cell did not express either of these markers. Those cells exhibiting an intermediate phenotype between the “recirculating” CD69-CD103- and CD69+CD103+ T_RM_ were classified as T_RM_-like (CD69+CD103-).[6,20,21]

**Figure 1:**
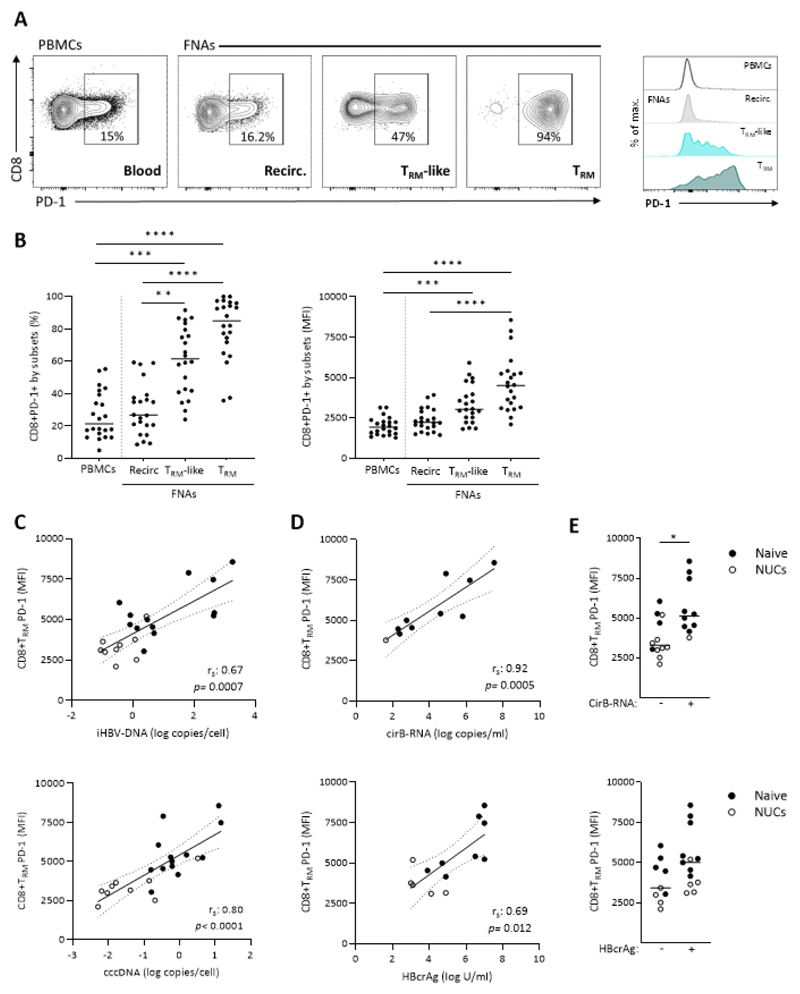
PD-1 expression on peripheral and intrahepatic CD8 T cells and its association with virological markers. (A) Representative dot plots and histograms showing the percentage and expression of PD-1 on circulating CD8 T cells and intrahepatic CD8 T cell subsets. Cells were pre-gated by CD45+CD3+CD56-CD8+. (B) Frequency of PD-1+ CD8 T cells and MFI (considering only PD-1+ CD8 T cells) in peripheral blood (PBMCs) and FNAs according to the expression of residency markers CD103 and CD69. Median values (black lines); Krustal-Wallis test and Dunn’s multiple comparisons test. Correlation between PD-1 MFI in T_RM_ and (C) iHBV-DNA and cccDNA, and (D) cirB-RNA and HBcrAg. Treated (NUCs) and untreated (Naive) patients are indicated by white and black dots, respectively. Linear regression (solid line) and 95% confidence bans of the best-fit line (dotted lines); Spearman correlation. (E) Expression of PD-1 on T_RM_ relative to detectable or undetectable levels of cirB-RNA and HBcrAg. Median values (black lines); Mann-Whitney U test. Phenotype of CD8 T cells according to the expression of CD69 and CD103: Recirc (recirculating CD69-CD103-, infiltrating but not tissue resident), T_RM_ (CD69+CD103+, tissue-resident memory) and T_RM_-like (CD69+CD103-, intermediate phenotype between recirc and T_RM_). In panels C, D and E, black dots represent naive patients, while white dots represent patients under NUC therapy. *p<0.05, **p<0.01, ***p<0.001, ****p<0.0001.

As previously reported, CD8 T_RM_ were not found in the periphery but were detectable in all FNAs, confirming reliable sampling of the intrahepatic compartment (T_RM_, CD69+ CD103+: 4%, IQR 2-7%; T_RM_-like, CD69+ CD103-: 20%, IQR 13-28%; Supplementary Figure S3A).[4] In addition, total CD8 T cells and NK cells were also enriched in the liver compartment whereas the frequency of B cells was lower in FNAs compared to blood (Supplementary Figure S3B). Likewise, the percentage and expression levels (MFI) of the coinhibitory receptor PD-1 was notably higher on intrahepatic than peripheral CD8 T cells, particularly in the tissue-resident fraction consisting of CD8 T_RM_ and the T_RM_-like populations (Figure 1A and B). Indeed, PD-1 MFI on CD8 T_RM_ was 2-fold higher than the non-resident subset (CD69-CD103-, p<0.0001), in line with previous observations.[4,6]

We then aimed to explore whether there was an association between viral activity and PD-1 expression on peripheral and intrahepatic CD8 T cells. We observed that the expression of PD-1 in CD8 T_RM_ (MFI) correlated with iHBV-DNA (r=0.67 p=0.0003) and cccDNA levels (r=0.80 p<0.0001; Figure 1C). These correlations were maintained when we separately analysed NUC-treated and untreated patients (data not shown). Moreover, these two markers also correlated with PD-1 expression in other subpopulations of T cells from FNAs but not in peripheral CD8 T cells (Supplementary Figure S4). Regarding serum viral markers, HBsAg levels did not correlate with PD-1 MFI in CD8 T_RM_. In contrast, we found a significant correlation between cirB-RNA and HBcrAg, the surrogate biomarkers of cccDNA transcriptional activity, and PD-1 expression on CD8 T_RM_ (Figure 1D). PD-1 expression in CD8 T_RM_ was also higher in patients with detectable cirB-RNA (90% untreated) as compared to those patients with undetectable cirB-RNA (67% of which were under NUC therapy, p=0.011). The same trend was observed for HBcrAg, though with no statistically significant differences (p=0.083; Figure 1E). Altogether, these data suggest an association between liver markers of HBV replication, serum markers of cccDNA activity and PD-1 expression on CD8 T_RM_.

### The expression of PD-1 on intrahepatic T cells is modulated by NUC therapy

**Figure 2:**
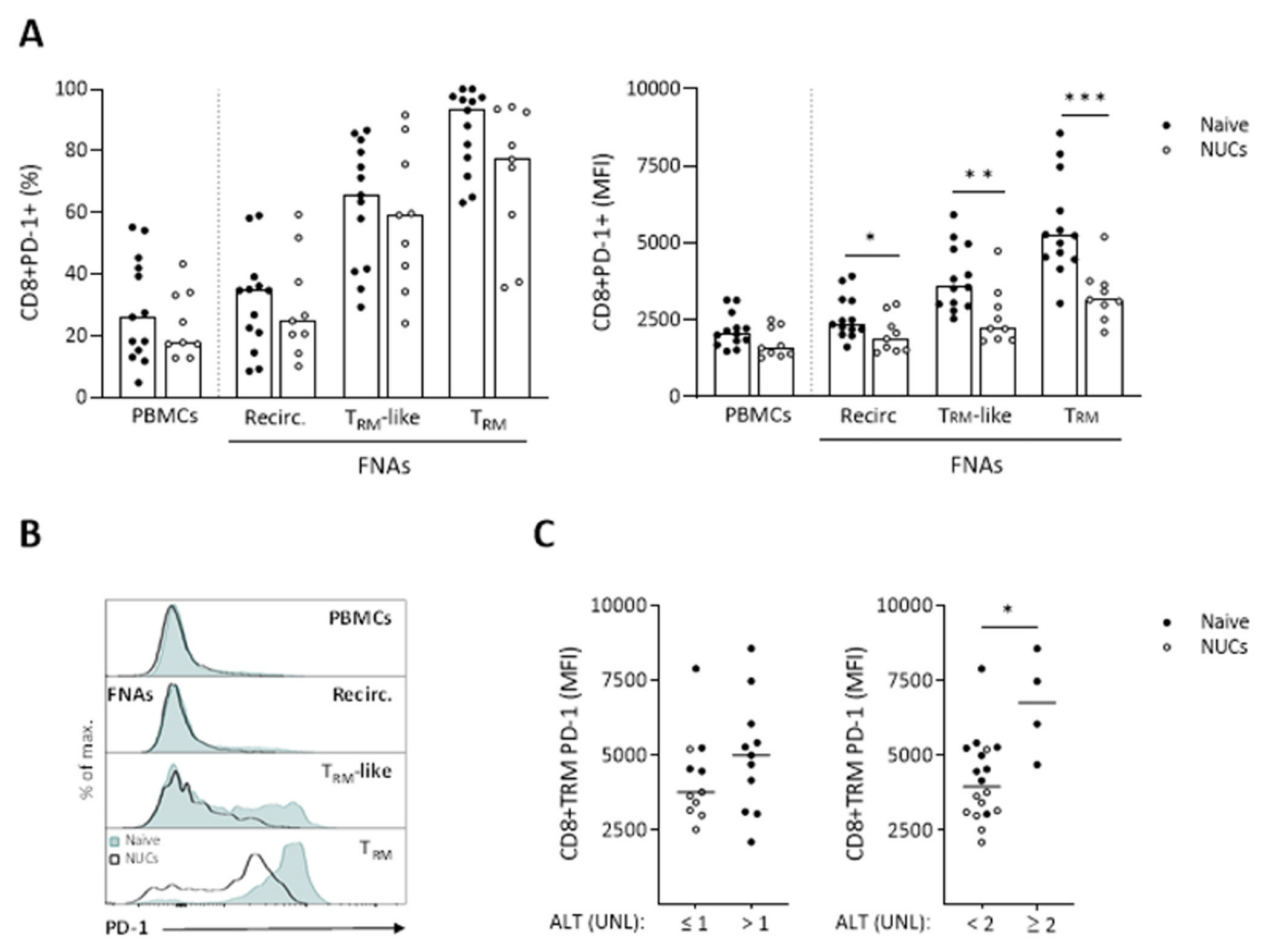
PD-1 expression on peripheral and intrahepatic CD8 T cells in naive versus NUC-treated patients. (A) PD-1 expression (percentage and MFI) on circulating and intrahepatic CD8 T cells subsets stratified based on residency markers. (B) Representative histogram plots depicting PD-1 expression. (C) Expression of PD-1 on T_RM_ according to ALT levels. Mann-Whitney U test Phenotype of CD8 T cells according to the expression of CD69 and CD103: Recirc (recirculating CD69-CD103-, infiltrating but not tissue resident), T_RM_ (CD69+CD103+, tissue-resident memory) and T_RM_-like (CD69+CD103-, intermediate phenotype between recirc and T_RM_). Bars and black lines indicate median values; black dots represent naive patients, while white dots represent patients under NUC therapy. *p<0.05, **p<0.01, ***p<0.001.

To investigate the impact of NUC treatment on the intrahepatic immune compartment, we classified the patients according to whether they were receiving NUC treatment or not. PD-1 expression (percentage and MFI) on liver-infiltrating CD8 T cells (CD69-CD103-) or circulating CD8 T cells was similar between both groups of patients. In contrast, we observed that PD-1 expression on CD8 T_RM_ and T_RM_-like was lower in patients on long-term NUCs than in naive/untreated patients (p=0.0008 and p=0.003; respectively; Figure 2A and B). When exploring whether these differences could be attributed to liver inflammation rather than viral activity, we observed that while median ALT levels were similar between the two groups (Table 1), PD-1 MFI on CD8 T_RM_ was increased in patients with higher ALT levels, all of whom were untreated (Figure 2C). Nonetheless, no significant correlation was found between PD-1 expression on CD8 T_RM_ and ALT levels in the subset of naive patients with mild ALT elevations (Supplementary Figure S5). These results suggest that phenotypic changes observed CD8 T cells residing in the liver are associated with HBV replication, even in presence of mild or subclinical inflammation.

**Figure 3:**
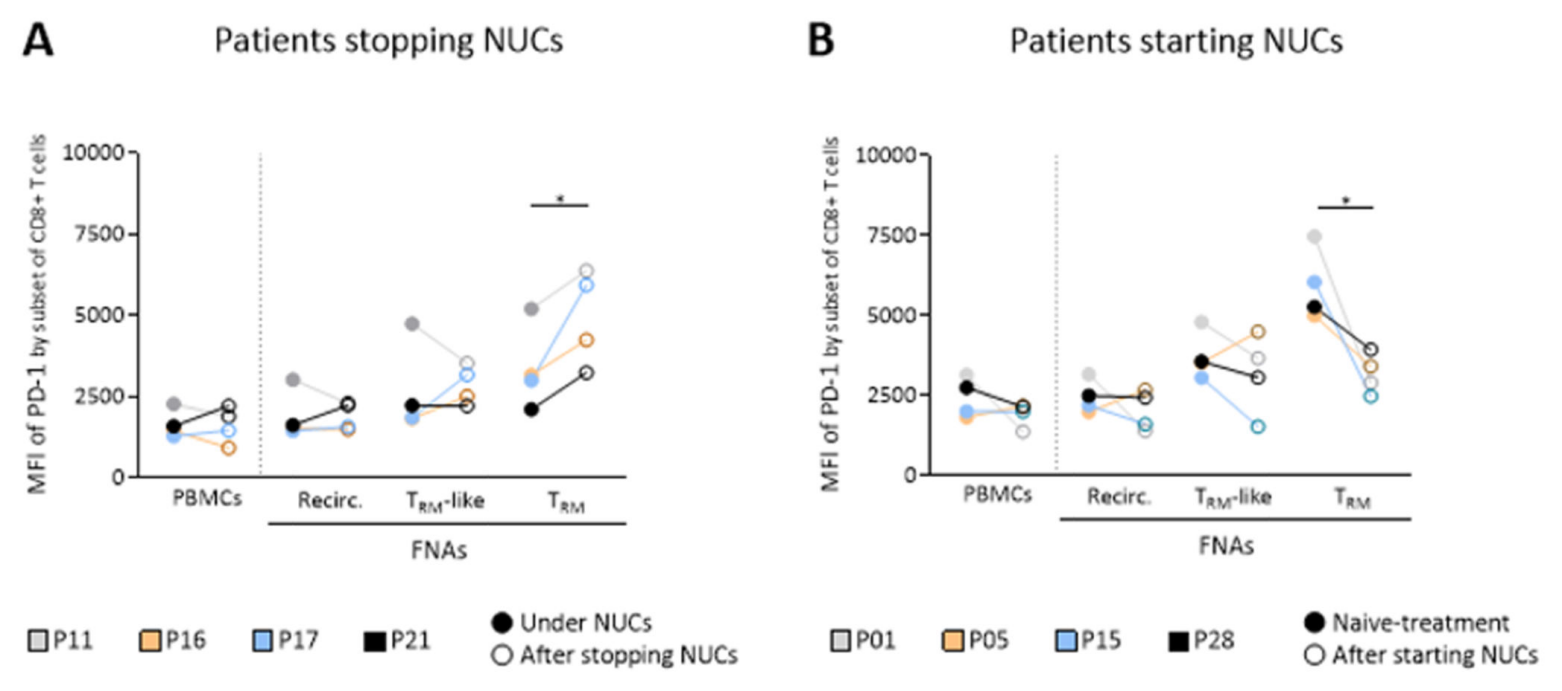
Immunological changes during follow-up after stopping or starting treatment with NUCs. Comparison of PD-1 expression (MFI) on CD8 T in PBMCs and FNAs between baseline and follow-up samples from (A) patients under NUCs therapy and 6 months after treatment discontinuation, and (B) treatment-naive patients and 1 year after starting antiviral therapy. CD8 T cells from FNAs are stratified based on the expression of residency markers CD69 and CD103: Recirc (recirculating CD69-CD103-, infiltrating but not tissue resident), T_RM_ (CD69+CD103+, tissue-resident memory) and T_RM_-like (CD69+CD103-, intermediate phenotype between recirc and T_RM_). Each colour represents a different patient. Paired t test. *p<0.05.

Cross-sectional differences between the NUC-treated and untreated groups could have been due to factors other than the effect of treatment on patient outcome. Thus, to further dissect a potential link with observed changes in intrahepatic PD-1 expression, we carried out longitudinal intra-patient comparisons before and after NUC interruption or introduction. Four HBeAg-negative CHB patients included in our study discontinued NUC therapy according to current EASL guidelines.[2] A follow-up FNA was performed in the absence of antiviral therapy in all patients. After a median follow-up of 13 months, one patient required treatment reintroduction according to previously defined criteria[22] while the other patients achieved viral control with low viral load and normal ALT. The kinetics of HBV-DNA, HBsAg and ALT during NUC discontinuation are shown in Supplementary Figure S6A. In all patients, HBV-DNA relapsed within the first 3 months after stopping NUCs and HBsAg levels remained positive at the time the follow-up FNA. Although all 4 patients experienced ALT elevations >1 ULN at some point after NUC discontinuation, ALT flares (defined as a rise in ALT >2 ULN) occurred in only 2. In comparison with baseline data, global lymphocyte populations (CD8 T, NK and B cells) did not change over the study period either in the periphery or in the liver (Supplementary Figure S7A). Interestingly, PD-1 expression on liver CD8 T_RM_ consistently increased after discontinuing NUCs, while it did not significantly vary on blood, recirculating, or T_RM_-like CD8 cells (Figure 3A).

Conversely, we re-sampled a subset of treatment-naive patients (n=4) between 9 and 18 months after starting NUC therapy (Supplementary Figure S6B). At the time of the follow-up FNA, HBV-DNA levels were <10 IU/ml and ALT <2 UNL in all patients. Again, no variations in the frequencies of the main immune populations were observed at follow-up (Supplementary Figure S7B). Remarkably, upon viral suppression and concomitant ALT decrease, PD-1 expression was downregulated on liver CD8 T_RM_, while no significant changes were observed in the other intrahepatic CD8+ T cell subsets or in the periphery (Figure 3B). Considering both groups of patients, we found a strong correlation between the percentage change before/after NUC initiation or discontinuation in PD-1 expression and ALT levels (r= 0.838, p= 0.013; Supplementary Figure S8). These results corroborate the previous observations from the cross-sectional comparison of naive and treated HBV patients, highlighting the association of NUC-induced modulation of HBV replication and liver inflammatory activity with intrahepatic T cell expression of PD-1.

### Single cell transcriptome profiling of CD8 T_RM_

**Figure 4:**
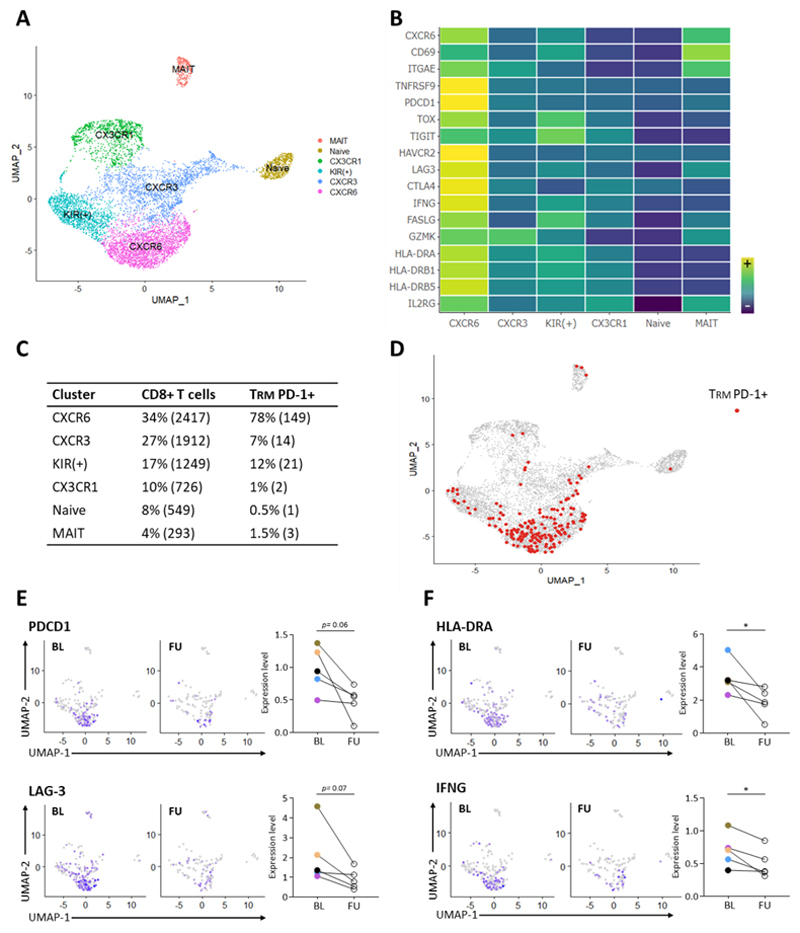
Single cell transcriptome profiling of CD8 T_RM_. (A) UMAP plot representation of CD8 T cell clusters named according to selected marker genes. CD8 T cells from all 5 patients starting NUC therapy (3 time points: baseline, week 12 and week 24). (B) Heatmap of the normalized expression level of the cell-type-specific genes in the CD8 T cell clusters. Color represents normalized expression level for each gene. (C) Percentage (and number) of CD8 T cells and PD-1+ CD8 T_RM_ in each cluster. (D) UMAP plot displaying PD-1+ CD8 T_RM_ cells highlighted (CD8+CD69+CD103+PD-1+) in red dots. Feature UMAP plots and single-cell gene expression for (E) exhaustion and (F) activation markers before (baseline, BL, full dots) and after 24 weeks of TAF therapy (follow-up, FU, open dots) for each patient. Paired t test. *p<0.05.

To further characterize the dynamic changes induced by NUC therapy in the individual liver immune cells and correlate them with our flow cytometry findings, we analyzed available FNA scRNA-seq data from a separate cohort of 5 CHB patients before and after 24 weeks of NUC therapy.[9] We identified 6 distinct CD8 T cell subpopulations that clustered according to the expression of differentially expressed selected marker genes (Figure 4A, 4B and Supplementary Figure S9). A prevalent tissue-resident population characterized by the expression of CXCR6 represented 34% of total CD8 T cells sampled in FNAs (Figure 4C). As recently reported by Nkongolo S et al.,[9] this CXCR6+ population also expressed the tissue residency markers CD69 and CD103 and was characterized by a high expression of both activation (TNFRSF9 [CD137/4-1BB], HLA-DR, IL2RG) and exhaustion (PDCD1 [PD-1], LAG3, TIGIT, HAVCR2 [TIM3], TOX) markers (Figure 4B). We identified CD8 T_RM_ cells in the FNA scRNAseq data using combined expression of CD69+CD103+PD-1+ and analysed their distribution across CD8 T cell clusters. We observed that 78% of this population overlapped with the CXCR6 cluster in the UMAP plot (Figure 4C and 4D). We visualized the CD8 T_RM_ on feature plots to analyse expression of key genes associated with activation and effector function. NUC-induced viral suppression and ALT normalization was associated with decreased expression of PD-1 RNA in CD8 T_RM_ at the single cell level (Figure 4E), consistent with decreased expression of PD-1 on this subpopulation at protein level by flow cytometry (Figure 2 and 3B). The expression of the inhibitory receptor LAG-3 on intrahepatic CD8 T_RM_ was also decreased (Figure 4E), while TOX was not consistently downregulated after NUC therapy (data not shown). Additionally, the levels of activation and effector markers such as HLA-DR and IFNG, respectively, were also reduced during follow-up (Figure 4F). These findings suggest that in patients with CHB, PD1+ CD8 T_RM_ cells may represent a bystander-activated population contributing to liver damage, and this phenotype could be modulated by NUC therapy.

## Discussion

Recent preclinical studies have added to the body of literature supporting multiple mechanisms of functional inhibition of T cells in CHB beyond the PD-1 pathway.[23–28] Nevertheless, PD-1 currently remains as a major investigational target in immunotherapeutic trials for functional cure of CHB, based on that existing data supporting the potential for PD-1 blockade to reinvigorate functionally impaired CD8+ T cells and/or boost therapeutic vaccine-induced responses in some present in patients with CHB.[13–16,29] To further understand the potential to target this checkpoint, we investigated the dynamics of PD-1 expression and its association with viral replication in treatment naive and NUC-treated CHB patients. Since there is a growing body of evidence suggesting that intrahepatic immune responses are not always mirrored in the periphery,[6,30,31] one of the main strengths of our study is that, in addition to blood samples, we utilized matched FNAs and biopsies to analyse the intrahepatic immune populations and liver viral biomarkers, respectively.[4,5,32,33] It is important to acknowledge that due to the small number of lymphocytes isolated from FNAs (two passes), we focused on the global CD8 T population to avoid biases stemming from insufficient numbers of intrahepatic HBV-specific T cells. While a significant proportion of HBV-specific CD8 T cells exhibit a resident phenotype,[6] the size and the activated profile of the PD1+CD8 T_RM_ subpopulation before treatment suggest that these cells include both HBV-specific and non-HBV-specific subsets. Notably, a recent study has demonstrated that the phenotype of total and HDV-specific CD8 T cells is similar in terms of the expression of inhibitory receptors and markers of activation and degranulation.[34]

In agreement with recent reports, we observed that PD-1 expression was strikingly higher in intrahepatic than circulating CD8 T cells, particularly the tissue-resident fraction T_RM_ (CD69+CD103+), and T_RM_-like (CD69+CD103-).[6,20] Interestingly, we show for the first time that the expression of PD-1 on CD8 T_RM_ correlated with both iHBV-DNA and cccDNA levels, but also with HBcrAg and cirB-RNA, serological biomarkers that reflect cccDNA transcriptional activity [19,35], in CHB patients with mild hepatitis. In contrast, no correlation was found between PD-1 expression on CD8 T_RM_ and HBsAg, likely because integrated HBV-DNA may constitute the primary source of HBsAg in our cohort (82% HBeAg-negative).[36,37] When evaluating the influence of ALT levels as a surrogate marker of liver damage in our treatment-naive cohort, composed mainly of patients with mild hepatitis, we did not observe a significant correlation between ALT levels and PD-1 expression on CD8 T_RM_. However, patients with elevated ALT levels (≥2 UNL) exhibited higher PD-1 MFI on CD8 T_RM_. These findings suggest that, in the context of subclinical inflammation, there is an association between HBV replication and PD-1 expression on CD8 T_RM_. A recent study, however, reported no correlation between intrahepatic immune activity, including PD-1 expression on T cells, and HBV parameters in untreated CHB patients.[38] This apparent discrepancy may be explained by differences in both patient characteristics and the T cell subsets analysed. While we specifically focused on CD8 T_RM_ in treatment-naive individuals with mild hepatitis, Van Buuren et al. examined total intrahepatic CD8 T cells from individuals with more active liver disease and a higher inflammatory burden. These factors could limit the ability to detect associations between PD-1 expression on CD8 T_RM_ and viral biomarkers. Additionally, intrahepatic markers of HBV replication such as cccDNA and iHBV-DNA were not assessed in that cohort, further limiting direct comparisons.

We also found significantly lower PD-1 expression on CD8 T_RM_ and T_RM_-like cells in NUC-treated patients compared to naive patients. To further investigate the impact of HBV replication in modulating PD-1 expression at the infection site, we conducted longitudinal sampling from a subset of patients after either starting or stopping antiviral NUC therapy. We observed that the expression of PD-1-on CD8 T_RM_ decreased in patients after starting NUCs, while no significant changes were observed in the periphery. Likewise, reinforcing these results, the analysis of those patients undergoing NUC discontinuation (resulting in HBV-DNA rebound and ALT elevation) demonstrated a pronounced increase in PD-1 expression in CD8 T_RM_, not reflected in recirculating non-infiltrating CD8 T cells and PBMC. These data support a role for HBV replication and/or accompanying changes in the milieu, driving PD-1 expression at the infection site.

Previous studies have shown that PD-1 expression decreased during NUC therapy on peripheral total and HBV-specific CD8 T cells of HBeAg-positive patients with a high viral load at baseline.[39,40] In our study, the percentage of PD-1-positive peripheral and recirculating CD8 T cells significantly decreased only in a HBeAg-positive patient (patient 1). This patient exhibited the most profound decline in viral load (>8 Log) and a 4-fold decrease in ALT levels after NUC therapy. Although we observed a correlation between the magnitude of PD-1 expression on peripheral and intrahepatic CD8 T cells (data not shown), the differences in PD-1 expression in the intrahepatic compartment between treated and naive patients were not reflected in peripheral CD8 T cells. Therefore, blood sampling cannot fully represent the impact of long-term viral suppression by NUCs on this immune target in the liver compartment in patients with mild ALT elevations, especially those with HBeAg-negative CHB. Indeed, in our cohort, we observed that, overall, NUC-induced HBV suppression was accompanied by a simultaneous decrease in ALT levels and PD-1 expression on CD8 T_RM_, while the reverse effect was observed with HBV-DNA relapse after NUC discontinuation. In agreement with previous observations, these data suggest that the inflammatory liver microenvironment, modulated by active HBV replication, is linked to upregulation of PD-1 on CD8 T_RM_ cells.[38]

To get a deeper insight into PD-1-expressing CD8 T_RM_, we analyzed intrahepatic longitudinal scRNA-seq data from 5 CHB patients initiating NUC therapy in a separate cohort.[9] Our PD-1+ CD8 T_RM_ population gated by flow cytometry as PD-1+CD69+CD103+, clustered together with a liver resident CXCR6+ polyclonal CD8 T cell population identified by scRNAseq. This population exhibited an activated signature despite the presence of exhaustion markers such as PDCD1, LAG3, TIM3, TIGIT and TOX. After 24 weeks of TAF treatment, the expression of effector molecules (IFNG and FASLG) and activation markers (HLA-DR, IL2RG) on CD8 T_RM_ decreased concomitantly with the expression levels of PD-1 and LAG3. As suggested by Nkongolo et al., this population may represent a hepatotoxic bystander CD8+ T cell population involved in CHB pathogenesis, mostly present when there is hepatocyte damage (elevated ALT levels). A similar population of liver-resident (CXCR6+) CD8 T cells, displaying both exhaustion and effector features, has been shown to contribute to liver immunopathology not only in chronic hepatitis delta but also in MAFLD, in an MHC class I independent manner.[34,41] In our cohort, with normal to mildly elevated ALT levels, the PD1+ CD8 T_RM_ population could reflect bystander activation of intrahepatic CD8 T cells in response to subclinical liver injury due to persistent HBV replication. Pathogen-associated molecular patterns (PAMPs) and damage-associated molecular patterns (DAMPs) generated during HBV replication may activate pattern recognition receptors (PRRs) in hepatocytes, inducing cytokine production that modulates PD-1 expression in intrahepatic T cells. Thus, a decrease in this population is consistent with the reduction in liver inflammation typically accompanying viral suppression induced by NUC treatment.

In conclusion, our data show an association between liver-resident T cell PD-1 expression and HBV replication together with a reduction by viral suppression through NUC therapy. The mechanism by which HBV load associates with global (rather than just HBV-specific) T cell PD-1 and how NUCs might modulate this remains to be explored. However, our findings have practical implications for the timing of ongoing trials of PD-1 blockade in relation to NUC treatment. Since PD-1 availability may be important for the response to anti-PD-1 immunotherapy [42,43], patients who have been on long-term NUCS may not respond optimally to this approach. Conversely patients stopping NUCs, with the attendant upregulation of PD-1, may have an improved response to PD-1 blockade, although this also might increase the risk of a liver flare [44]. Future studies should similarly investigate the impact of the new generation of antivirals able to reduce viral antigens on T cell PD-1. By sampling compartmentalised liver T cells, our results provide new insights into the impact of NUCs on a current immunotherapeutic target on the path to functional cure.

## Supplementary Material

Supplementary Material

## Figures and Tables

**Table 1:** Baseline clinical and virological characteristics of patients according to treatment status (treatment-naive or NUC-treated).

	Treatment-naive (n=13)	NUC-treated (n=9)	p value
Sex (male)	10 (77%)	6 (67%)	n.s.
Age (years)	44 (34 - 58)	47 (42 - 58)	n.s.
ALT (U/L)	41 (32 - 87)	25 (22 - 41)	n.s.
Fibrosis stage			
F0-1	10 (77%)	9 (100%)	
F2/F3	1/2 (8%/15%)		n.s.
Treatment			
TDF/ETV	n.a.	7/2 (78%/22%)	n.a.
Duration (years)		7(5 - 10)	
HBeAg (-)	8 (62%)	9 (100%)	0.03
**Serum markers**			
HBV-DNA (Log lU/ml)	5.2 (3.4 - 8.4)	n.a.	n.a.
HBsAg (Log lU/ml)	4.1 (3.1 - 4.4)	3.3 (2.5 - 4.1)	n.s.
HBcrAg_cat (positive)	8 (62%)	5 (56%)	n.s.
HBcrAg (Log U/ml)*	6.6 (4.8 - 7.0)	3.1 (3.1 - 4.5)	0.01
cirB-RNA_cat (positive)	9 (69%)	1 (11%)	0.007
cirB-RNA (Log copies/ml)*	4.6 (2.6 - 6.0)	n.a.	n.a.
**Liver markers**			
iHBV-DNA (copies/cell)	4.39 (1.10 - 426)	0.28 (0.10 - 0.78)	0.0026
cccDNA (copies/cell)	0.61 (0.30 - 3.00)	0.02 (0.008 - 0.17)	0.0011

Categorical and quantitative data are expressed as n (%) and median (IQR), respectively. Groups were compared using the Chi-square test for categorical variables and the Mann-Whitney U test for continuous variables.

* Median (IQR) values for HBcrAg and cirB-RNA were calculated considering only the detectable values. n.s., non-significant; n.a., non-available.

## Data Availability

Information about patients included in the study are confidential. Additional data relevant to readers is provided as supporting information.

## Lay Summary

PD-1 is a major investigational target in current immunotherapies for chronic hepatitis B. Our results reveal a close association between HBV replication and the expression of PD-1 on liver-resident T cells but not on circulating lymphocytes. Therefore, deeper understanding of the dynamics of PD-1 expression on immune cells may facilitate the optimization of treatment strategies that incorporate immunomodulatory agents, advancing us toward the goal of achieving a functional cure.

## Abbreviations

CHB: Chronic hepatitis B
NUC: nucleos(t)ide analogs
FNAs: fine needle aspirate
T_RM_: tissue-resident memory T cells
scRNAseq: single cell RNA sequencing
PBMC: peripheral blood mononuclear cells
HBcrAg: HBV core-related antigen
cirB-RNA: circulating HBV-RNA
cccDNA: total intrahepatic HBV-DNA, iHBV-DNA, covalently-closed circular DNA
MFI: mean fluorescence intensity
