# Supplementary material for: HBV suppression by nucleos(t)ide analogues reduces PD-1 expression on liver-resident T cells
Source: Liver Int. Author manuscript; Available in PMC 2026 Aug 12. (PMC7619358; doi:10.1111/liv.70338)
Supplement: Supplementary file 1 [file EMS216193-supplement-Supplementary_Material.pdf]

ONLINE SUPPORTING INFORMATION

HBV suppression by nucleos(t)ide analogues reduces PD-1 expression on liver-resident T cells

Mireia García-López†[1], Sabela Lens†[1], Laura J. Pallett[2], Anna Pocurull[1], Thais Leonel[1], Ernest Belmonte[3], Ester García-Pras[1], Sergio Rodríguez-Tajes[1], Zoe Mariño[1], Maria Sàez-Palma[1], Concepción Bartres[1], Ariadna Rando-Segura[4], Francisco Rodríguez-Frías[4], Jonah Li[5 6], Adam J Gehring[5 6], Mala K. Maini[2], Xavier Forns*[1], Sofía Pérez-del-Pulgar*[1].

Affiliations

1. Liver Unit, Hospital Clínic, University of Barcelona, IDIBAPS, CIBEREHD, Barcelona, Spain.
2. Institute of Immunity and Transplantation, Division of Infection and Immunity, University College London, London, UK.
3. Radiology Department, Centro de Diagnóstico por la Imagen (CDI), Hospital Clínic, University of Barcelona, Barcelona, Spain.
4. Liver Pathology Unit, Department of Biochemistry and Microbiology, Hospital Universitari Vall d'Hebron, Universitat Autònoma de Barcelona, CIBERehd, Barcelona, Spain.
5. Toronto Centre for Liver Disease, Toronto General Hospital Research Institute, University Health Network, Toronto, Ontario, Canada.
6. Department of Immunology, University of Toronto, Toronto, Ontario, Canada.

Tables and Figures 2

Table S1. List of antibodies used in flow cytometry analysis. 2

Table S2. Individual patients' characteristics. 3

Figure S1. Correlations between the different viral biomarkers. 4

Figure S2. Flow cytometry gating strategy used to identify lymphocyte populations in a representative FNA sample. 5

Figure S3. Global lymphocyte profiling of matched blood (PBMCs) and liver FNA samples. ... 6

Figure S4. Correlations between PD-1 expression in CD8 T cell subsets and viral markers of HBV replication. 7

Figure S5. Viral kinetics after treatment discontinuation or initiation. 8

Figure S6. Global lymphocyte profiling of matched blood and liver FNAs at baseline and during follow-up. 9

Figure S7. Correlation between the percentage change in PD-1 expression and ALT levels during patient follow-up. Spearman correlation. 9

Figure S8. The six clusters for CD8 T cells and expression of feature genes. 10

Tables and Figures

Table S1. List of antibodies used in flow cytometry analysis.

Specificity	Fluorochrome	Cat. No.	Manufacturer	Isotype	Clone
CD3	BUV805	565515	BD	IgG1	UCHT1
CD45	BUV395	563792	BD	IgG1	HI30
CD19	BV786	563325	BD	IgG1	SJ25C1
CD69	BV711	310944	Biolegend	IgG1	FN50
CD103	BV605	350218	Biolegend	IgG1	Ber-ACT8
IFN-y	V500	561980	BD	IgG1	B27
IL-2	Pacific Blue	500324	Biolegend	Rat IgG2a	MQ1-17H12
CD107a	BB700	566558	BD	IgG1	H4A3
CD56	PE-Cy7	335826	BD	IgG2b	NCAM16.2
CD20	PE-Dz	302348	Biolegend	IgG2b	2H7
L/D	APC-Cy7	L34975	ThermoFisher	-	-
CD8	Alexa Fluor 700	56-0086-42	eBioscience	IgG2a	OKT8
PD-1	APC	329908	Biolegend	IgG1	EH12.2H7

Table S2. Individual patients' characteristics.

Patient ID	Age (years)	Sex	Therapy	Genotype	HBeAg	Serum HBV-DNA (IU/ml)	HBsAg (IU/ml)	HBcrAg (Log U/ml)	cirB-RNA (copies/ml)	ALT (U/L)	iHBV-DNA (copies/cell)	cccDNA (copies/cell)	Fibrosis stage
P01	44	M	No	A	Positive	537000000	62147	7.0	1550000	195	420.3	14.486	0
P02	37	F	No	A	Negative	2920	24990	UND	UND	14	2.29	0.160	0
P03	34	M	No	D	Negative	155000	14641	4.9	238	48	4.88	0.901	0
P05	44	F	No	C	Negative	220000	11882	4.7	565	30	2.96	0.606	0
P07	26	M	No	E	Positive	565000000	83691	7.0	642000	34	432.6	4.432	0
P09	24	F	No	C	Positive	1000000000	27971	7.0	33000000	684	1800.5	12.713	3
P12	62	M	No	B	Negative	48400	322	UND	195	21	1.37	0.155	0
P15	34	M	No	C	Negative	2190	1087	UND	UND	82	0.36	0.252	0
P18	47	M	No	F	Negative	2090	3913	UND	UND	91	0.04	0.613	0
P23	54	M	No	C	Positive	14600000	1223	6.5	39800	41	455.7	1.551	3
P25	68	M	No	B	Negative	166000	1545	3.9	1080	34	4.39	0.338	2
P27	67	M	No	B	Negative	669000	1031	6.7	78400	40	65.44	0.342	1
P28	48	M	No	E	Negative	959	12476	UND	UND	45	0.82	0.560	1
P04	63	M	Entecavir	D	Negative	UND	179	4.1	UND	43	0.09	0.006	1
P11	66	M	Tenofovir	A	Negative	UND	17987	3.1	UND	20	2.75	3.225	0
P14	44	M	Tenofovir	C	Negative	UND	521	3.1	UND	25	0.10	0.016	0
P16	43	F	Tenofovir	B	Negative	UND	4556	4.9	UND	25	0.29	0.041	0
P17	41	M	Tenofovir	E	Negative	UND	2916	UND	UND	39	0.12	0.009	0
P20	50	F	Entecavir	F	Negative	UND	23378	UND	UND	32	0.04	0.203	0
P21	41	M	Tenofovir	D	Negative	UND	2227	UND	UND	45	0.28	0.005	0
P24	47	M	Tenofovir	B	Negative	UND	860	3.0	41.3	23	1.18	0.138	1
P26	53	F	Tenofovir	-	Negative	UND	9	UND	UND	18	0.37	0.012	1

Undetectable levels of serum HBV-DNA, HBcrAg and cirB-RNA are shown as UND (below LOD/LOQ).

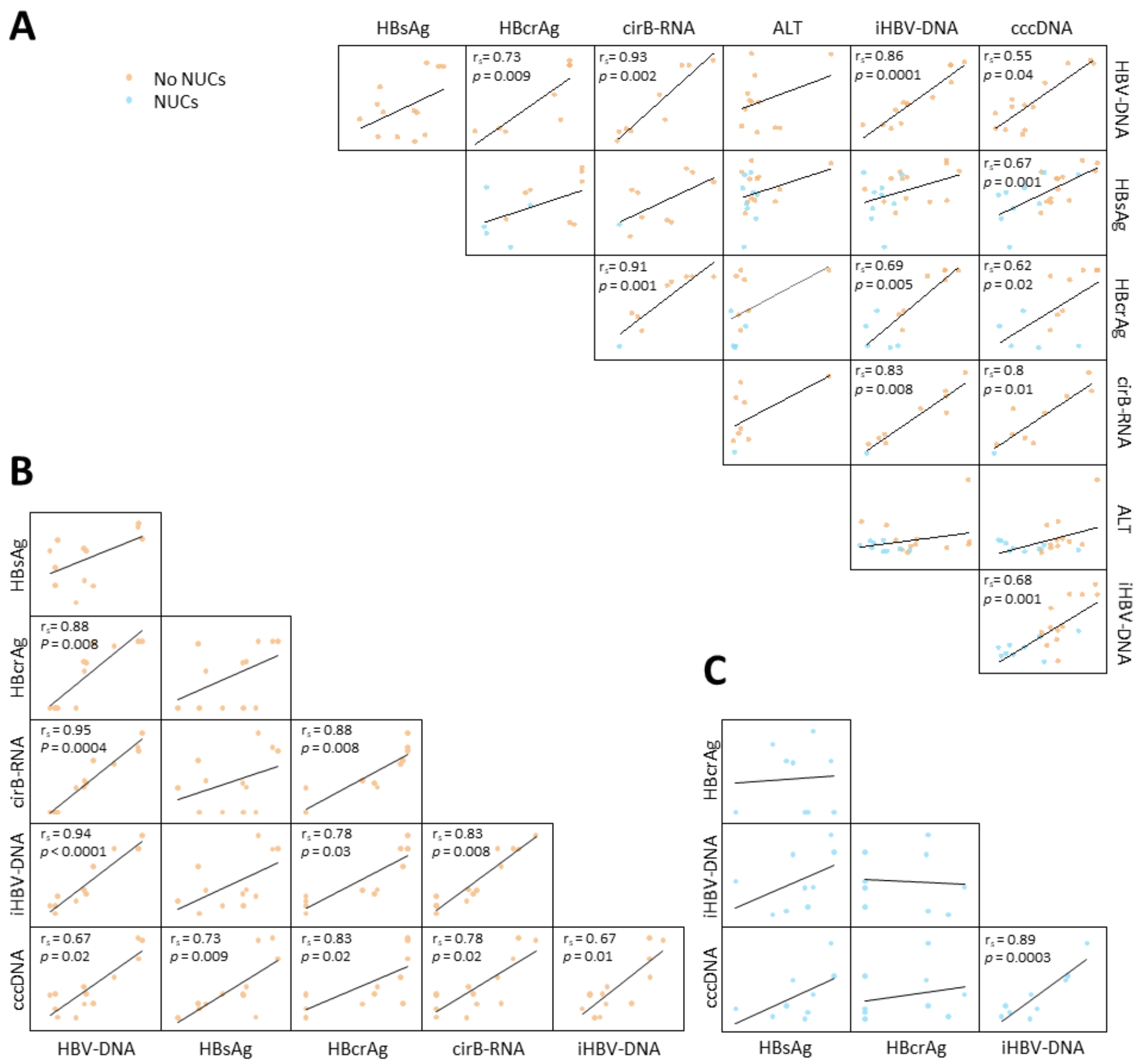

Figure S1. Correlations between the different viral biomarkers. (A) All patients, (B) Naive-treated patients, and (C) Patients treated with NUCs. In case of undetectable values, patients were excluded from the corresponding analysis. Spearman's correlation.

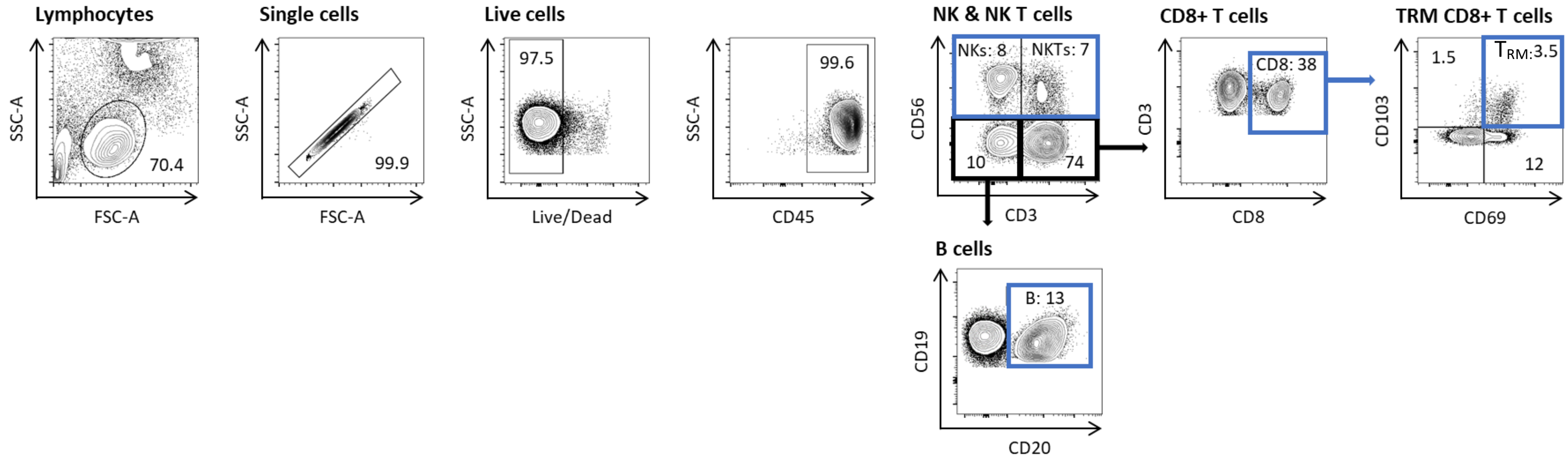

Figure S2. Flow cytometry gating strategy used to identify lymphocyte populations in a representative FNA sample.

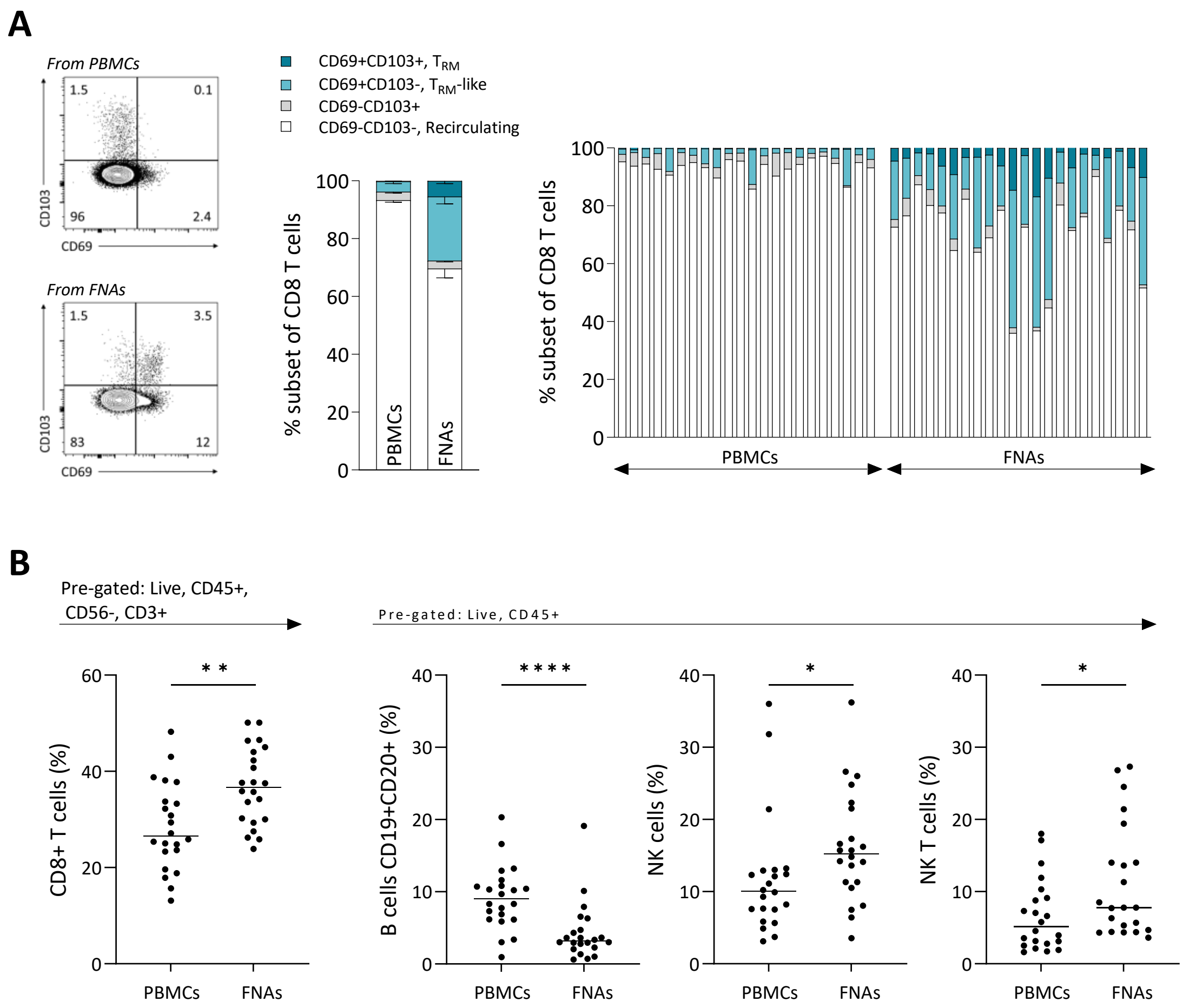

Figure S3. Global lymphocyte profiling of matched blood (PBMCs) and liver FNA samples. (A) Identification of liver resident CD8 T cells (T_{RM}, CD69+CD103+), T_{RM}-like (CD69+CD103-) and non-resident tissue-infiltrating "recirculating" (CD69-CD103-). Stacked bar charts show the frequency of each subset mean with SEM (left plot), and individual data where each bar corresponds to one patient (right plot). (B) Global CD8 T cells, B cells, NK cells and NK T cells in PBMCs and FNAs. Mann Whitney U test.

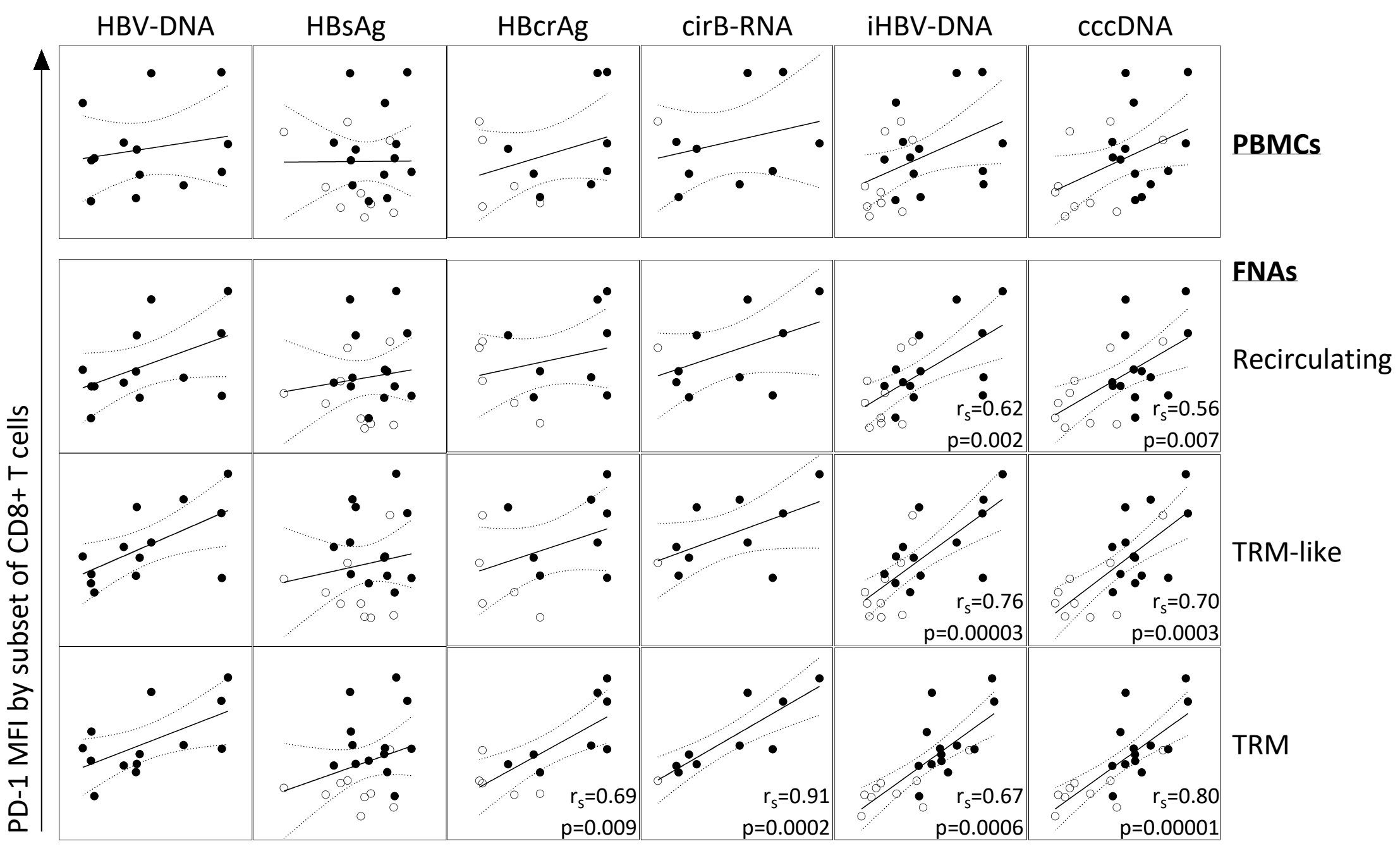

Figure S4. Correlations between PD-1 expression in CD8 T cell subsets and viral markers of HBV replication. Black and white dots represent naive and treated patients, respectively. Spearman correlation.

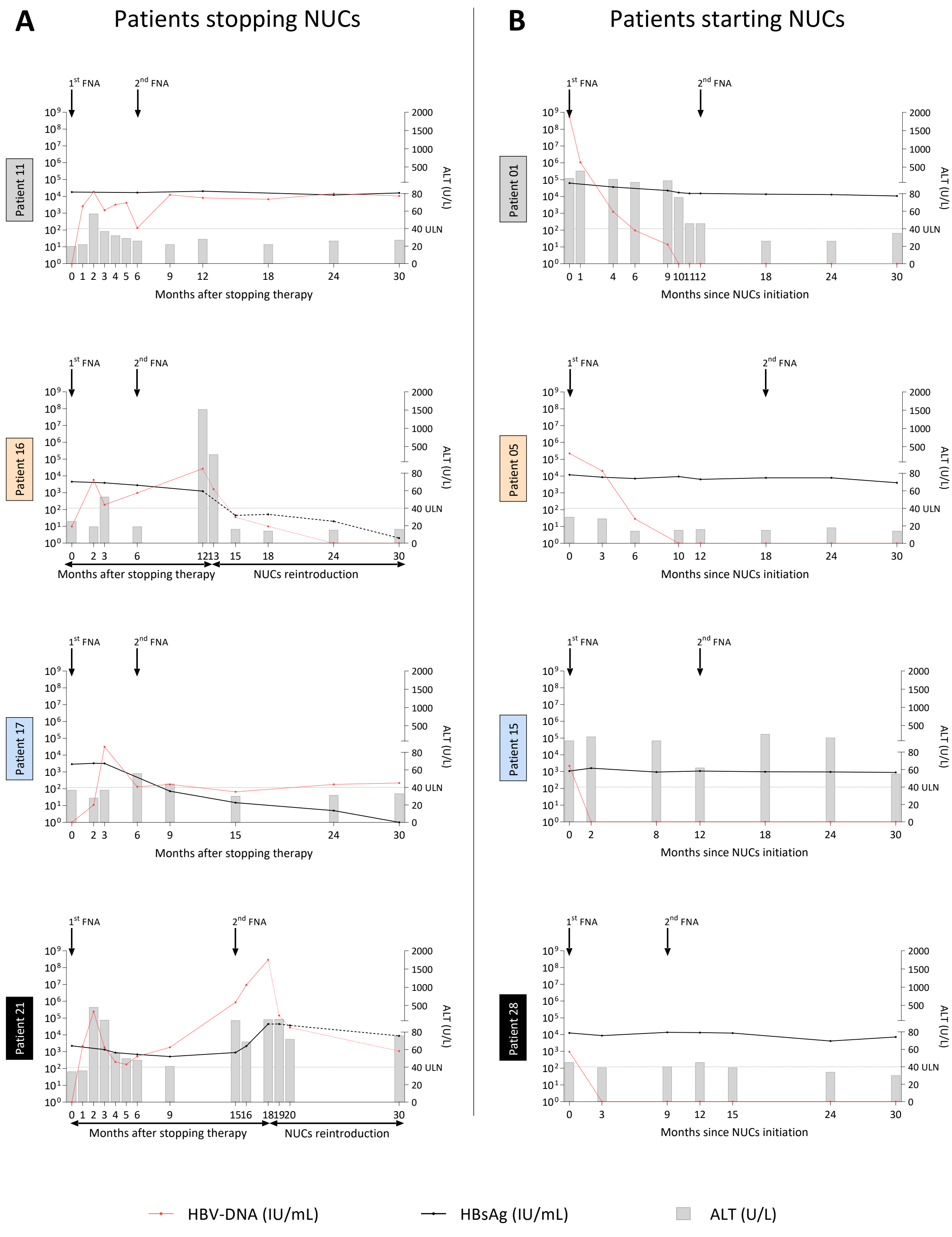

Figure S5. Viral kinetics after treatment discontinuation or initiation. Longitudinal analysis of HBV-DNA, HBsAg, and ALT kinetics in A) 4 patients undergoing NUC discontinuation and B) 4 patients starting NUC treatment. * Note that in P05, the 1st FNA was performed 1 year before starting NUCs

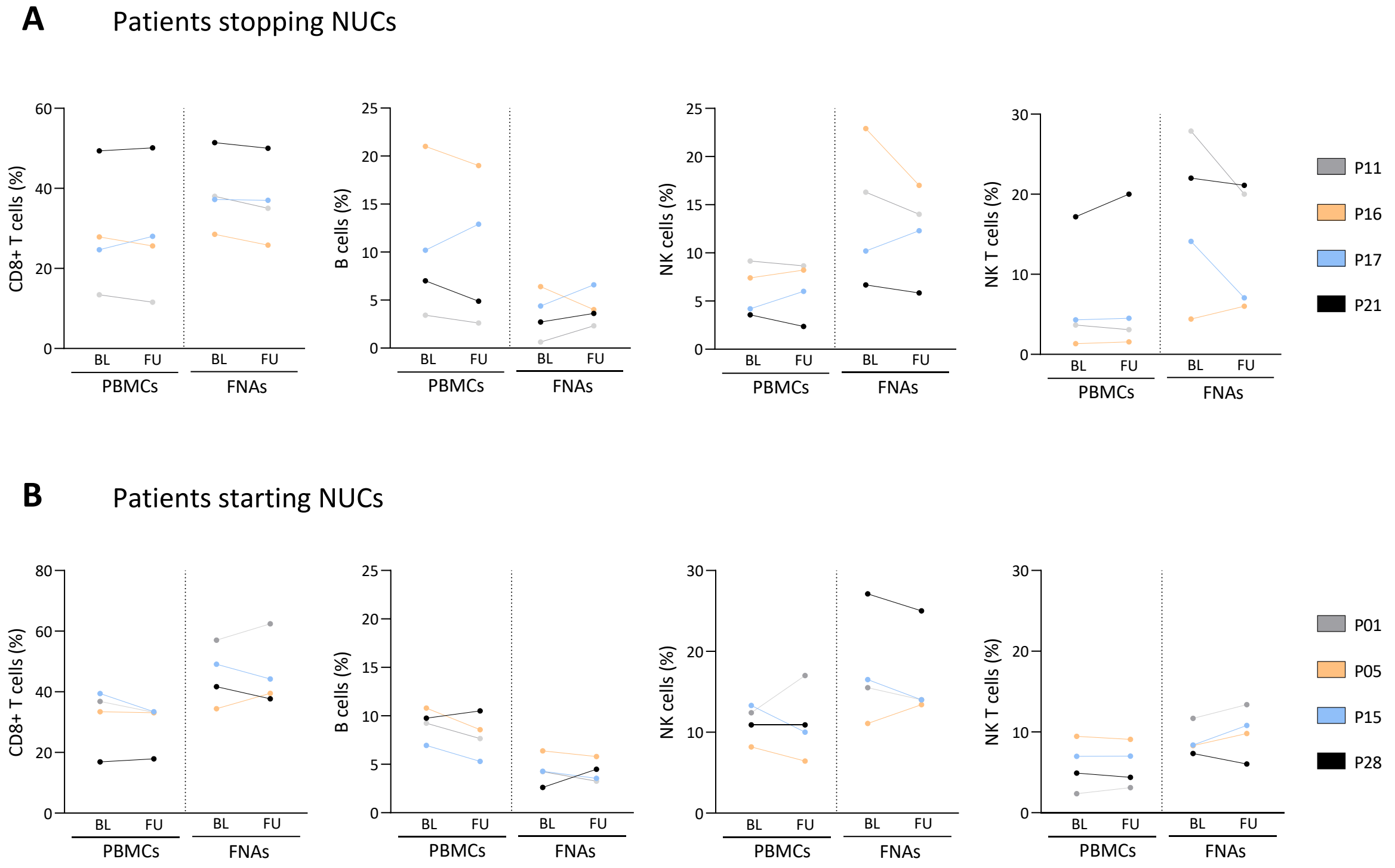

Figure S6. Global lymphocyte profiling of matched blood and liver FNAs at baseline and during follow-up. A) Patients who underwent NUC withdrawal, and B) patients who started NUC treatment. Paired t-test. BL, baseline; FU, follow-up.

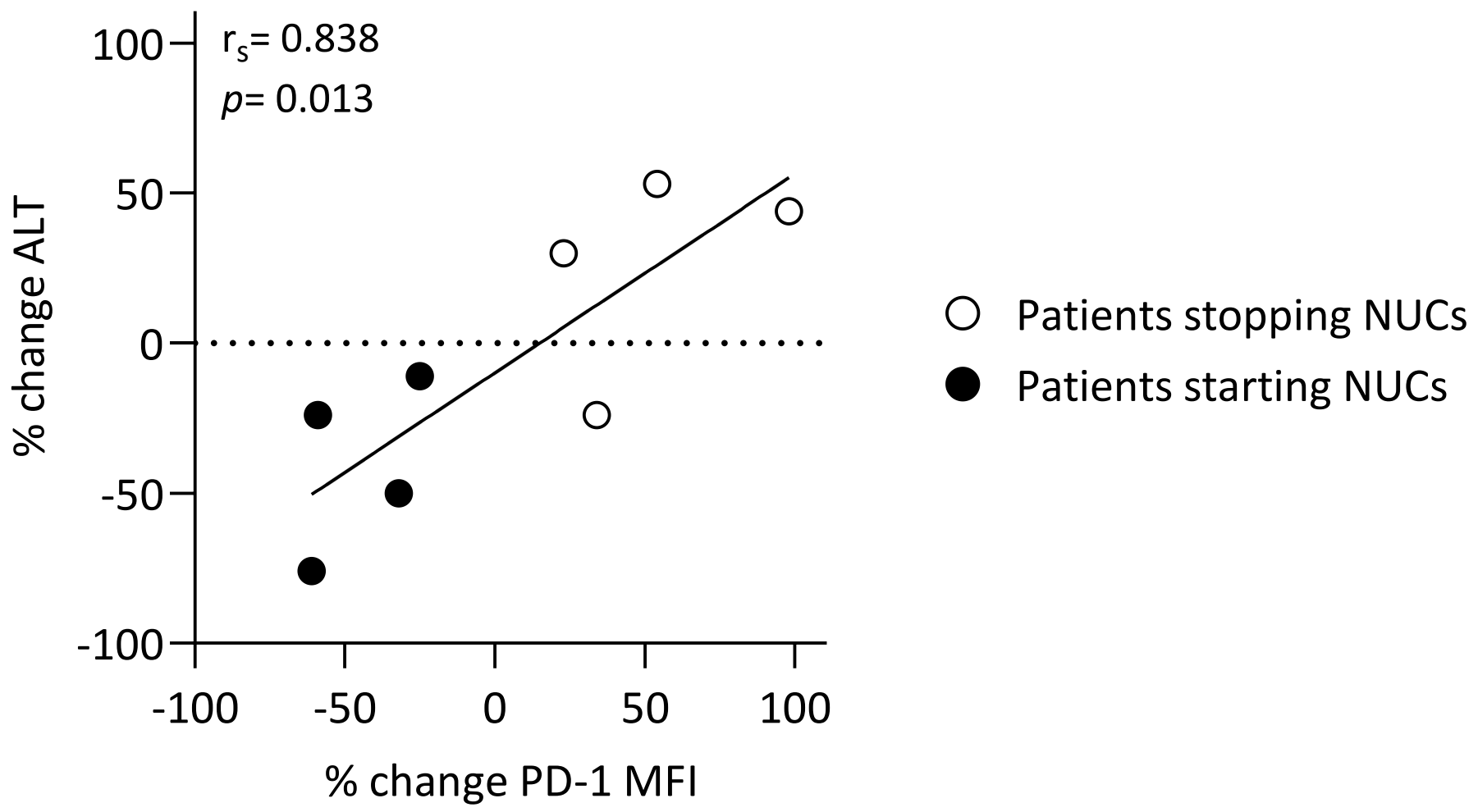

Figure S7. Correlation between the percentage change in PD-1 expression and ALT levels during patient follow-up. Spearman correlation.

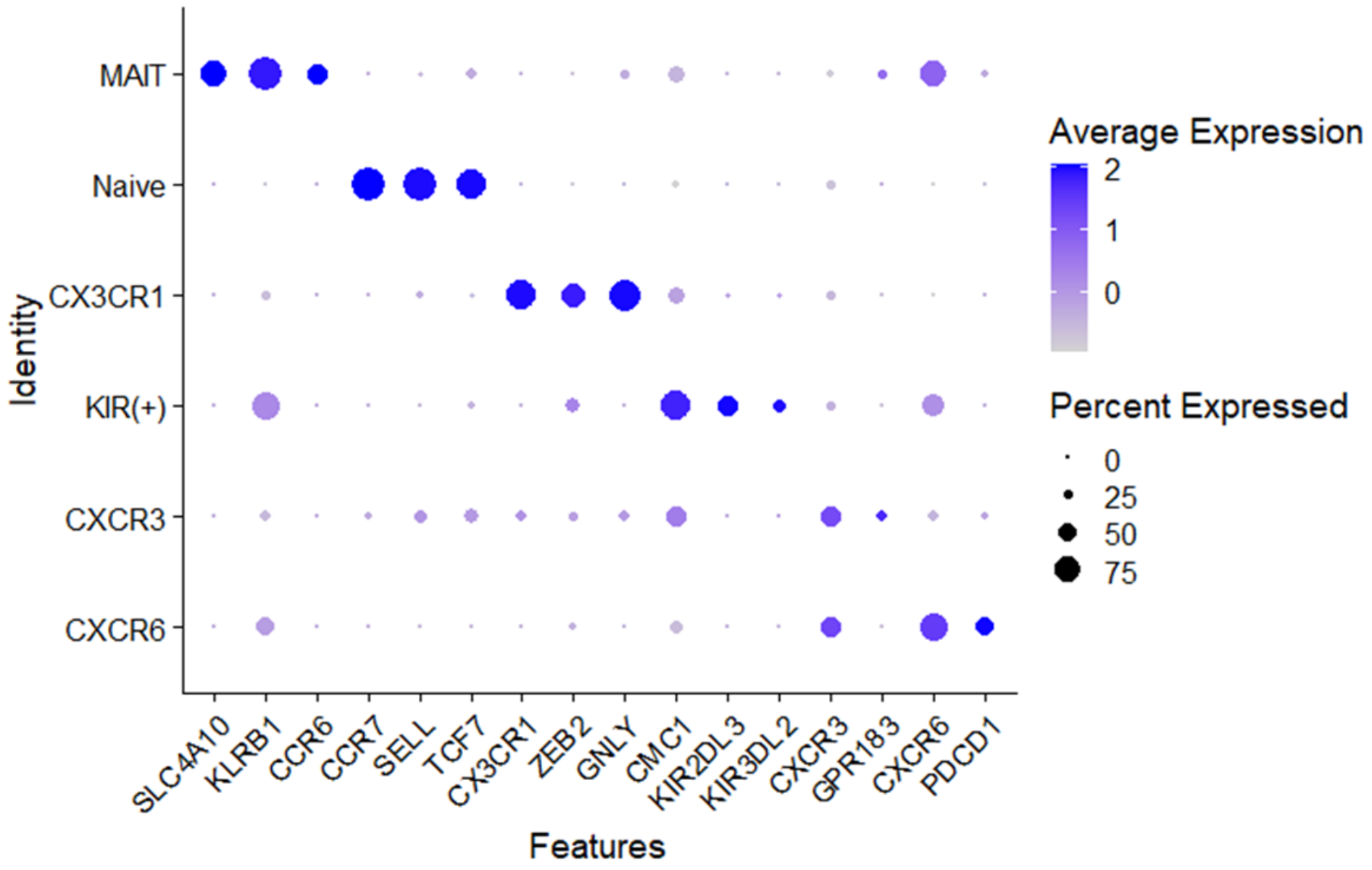

Figure S8. The six clusters for CD8 T cells and expression of feature genes. Cell types were annotated based on the canonical marker gene expressions. Colour intensity represents the normalized expression level and dot size the percentage of cells within each cluster expressing the corresponding marker gene.
